# Isoflavones daidzin and daidzein inhibit lipopolysaccharide-induced inflammation in RAW264.7 macrophages

**DOI:** 10.1186/s13020-022-00653-0

**Published:** 2022-08-16

**Authors:** Yi Tan, Xutao Zhang, Wai San Cheang

**Affiliations:** State Key Laboratory of Quality Research in Chinese Medicine, Institute of Chinese Medical Sciences, University of Macau, Macao SAR, China

**Keywords:** Daidzin, Daidzein, Inflammation, Lipopolysaccharides, Macrophages

## Abstract

**Background:**

Inflammation contributes to various diseases and soybeans and legumes are shown to reduce inflammation. However, the bioactive ingredients involved and mechanisms are not completely known. We hypothesized that soy isoflavones daidzin and daidzein exhibit anti-inflammatory effect in lipopolysaccharides (LPS)-stimulated RAW264.7 macrophage cell model and that activation mitogen-activated protein kinase (MAPK) and nuclear factor kappa B (NF-κB) signaling pathways may mediate the effect.

**Methods:**

Cell viability and nitric oxide (NO) level were determined by 3-(4,5)-dimethylthiazol-2-yl)-2,5-diphenyltetrazolium bromide (MTT) assay and Griess reagent respectively. ELISA kits and Western blotting respectively assessed the generations of pro-inflammatory cytokines and protein expressions of signaling molecules. p65 nuclear translocation was determined by immunofluorescence assay.

**Results:**

The in vitro results showed that both isoflavones did not affect cell viability at the concentrations being tested and significantly reduced levels of NO, pro-inflammatory cytokines such as interleukin (IL)-6 and tumor necrosis factor-α (TNF-α), and inflammatory indicators such as cyclooxygenase-2 (COX-2) and inducible nitric oxide synthase (iNOS) in RAW264.7 cells. Daidzin and daidzein partially suppressed MAPK signaling pathways, reducing the phosphorylation of p38 and ERK; whilst phosphorylation of JNK was mildly but not significantly decreased. For the involvement of NF-κB signaling pathways, daidzin only reduced the phosphorylation of p65 whereas daidzein effectively inhibited the phosphorylation of IKKα/β, IκBα and p65. Daidzin and daidzein inhibited p65 nuclear translocation, comparable with dexamethasone (positive control).

**Conclusion:**

This study supports the anti-inflammatory effects of isoflavones daidzin and daidzein, which were at least partially mediated through inactivation of MAPK and/or NF-κB signaling pathways in macrophages.

## Background

**Fig. 1:**
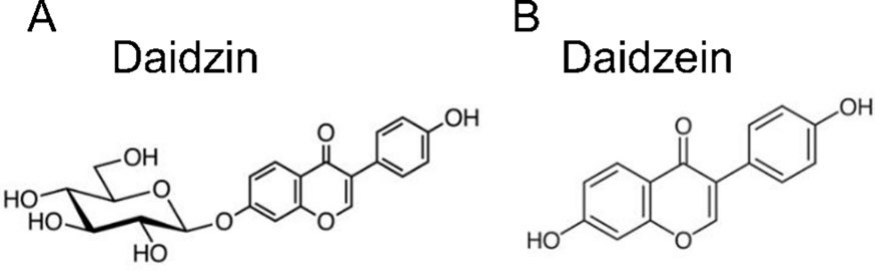
Chemical structural formula of daidzin and daidzein. **A** Daidzin and **B** daidzein structure

Daidzin (7-(β-d-glucopyranosyloxy)-4′-hydroxyisoflavone) and daidzein (4′,7-dihydroxyisoflavone) are major isoflavones found in soybeans and other legumes; and are also present in *Radix Puerariae* (Gegen) which is a herbal medicine prepared from the root of leguminous *Pueraria lobata* plants [1]. In traditional Chinese medicine, soybeans are good for nourishing the spleen and dissipating dampness; whilst Gegen promotes the production of body fluid, relieves thirst and dispels wind evil [2]. Daidzin is the corresponding glucoside form of daidzein with their structures illustrated in Fig. 1A, B, and Daidzin can release daidzein through hydrolysis [3, 4]. As a member of soy isoflavones, daidzin and daidzein are phytoestrogens that mimic estrogen in the body and are beneficial for women’s health [5, 6]. According to previous studies, daidzin and daidzein can protect against osteoporosis [7–9], cardiovascular and cerebrovascular diseases [10–12].

Many diseases are accompanied by inflammation, such as cardiovascular disease [13], diabetes [14, 15], cancer [16, 17] and arthritis [18]; and thereby effective means to modulate systemic inflammation is very important. Such inflammatory responses generally involve the activation of mitogen-activated protein kinase (MAPK) and/or nuclear factor kappa B (NF-κB) signaling pathways. Daidzein has been reported to possess anti-inflammatory activity in different models by previous studies. Daidzein ameliorates lipopolysaccharides (LPS)-induced inflammation in the hepatocytes [19]. Moreover, daidzein has anti-inflammatory action in tumor necrosis factor-α (TNF-α)-treated murine lung epithelial cells [20] and in TNF-α-stimulated Caco-2 cells [21]. Daidzein also suppresses inflammation in activated macrophages upon LPS exposure by inhibiting the signal transducer and activator of transcription 1 (STAT-1) and NF-κB activations [22, 23]. On the other hand, there is limited study on the anti-inflammatory effect of daidzin. Daidzin has been demonstrated to inhibit LPS-induced nitric oxide (NO) generation from macrophages [24]; nevertheless, the underlying mechanism is not recognized and needs to be explored. Importantly, whether daidzein may act on other signaling pathway such as the MAPK pathway remains to be investigated, apart from the NF-κB signaling cascade shown in previous studies. We hypothesized that daidzin and daidzein can exert anti-inflammatory effect in LPS-stimulated RAW264.7 macrophages and that MAPK and/or NF-κB signaling pathways will be the crucial regulatory pathway.

## Methods

### Chemicals and reagents

Daidzin (7-(β-d-glucopyranosyloxy)-4′-hydroxyisoflavone) and daidzein (4′,7-dihydroxyisoflavone) were purchased from Tokyo Chemical Industry Co. Ltd. (Tokyo, Japan). LPS (*Escherichia coli* O111:B4), Griess reagent and 3-(4,5-dimethylthiazol-2-yl)-2,5-diphenyltetrazolium bromide (MTT) reagent were obtained from Sigma-Aldrich (St. Louis, MO, USA). All the primary antibodies used in this study were purchased from Cell Signaling Technology (Beverly, MA, USA); while the secondary antibodies and enzyme-linked immunosorbent assay (ELISA) kits for interleukin-6 (IL-6) and TNF-α were acquired from Beyotime Biotechnology (Shanghai, China).

### Cell culture

Mouse RAW264.7 macrophages were obtained from American Type Culture Collection (Rockville, MD, USA). The cells were cultured in Dulbecco’s modified eagle medium DMEM/HIGH GLUCOSE medium (GE Healthcare Life Sciences HyClone Laboratories, Utah, USA) containing 1% penicillin–streptomycin (Gibco, GrandIsland, NY, USA) and 10% Fetal bovine serum (FBS; Gibco). Then, the cells were maintained at 37 °C in a 5% CO_2_ humidified incubator.

### Cell viability assay

The cytotoxic effects of daidzin and daidzein were measured by the MTT assay. RAW264.7 cells (7 × 10^3^ cells/well) were seeded into 96-well culture plates and cultured overnight (5% CO_2_, 37 °C). Afterward, the cells were treated with different concentrations (10, 30, 50 and 100 µM) of daidzin and daidzein for 24 h. Medium with 10% MTT was added into each well to be incubated for 3 h. Finally, the supernatants were discarded and DMSO (150 µL) was added to each well for 30 min to dissolve the formazan crystals. The absorbance was measured at 570 nm by using SpectraMax M5 microplate reader (Molecular Devices, Silicon Valley, CA, United States).

### Quantification of NO release

Griess reagents kit was used to evaluate the production of NO. RAW264.7 cells (1 × 10^4^ cells/well) were seeded into 24-well culture plates and cultured overnight (5% CO_2_, 37 °C). The cells were pretreated with different concentrations (10, 30, 50 and 100 µM) of daidzin and daidzein for 4 h, followed by LPS (1 µg/mL) stimulation for 12 h to cause cell inflammation. Nitrite in the medium was determined with Griess reagent according to the manufacturer’s instructions. The absorbance was measured at 548 nm by using a microplate spectrophotometer reader.

### ELISA

RAW264.7 macrophages (6 × 10^5^ cells/well) were seeded into 6-well plates and incubated overnight (5% CO_2_, 37 °C), followed by drug treatment of daidzin and daidzein (50 µM and 100 µM pretreated for 4 h) with LPS stimulation (1 µg/mL, 12 h). The conditioned culture medium was collected to determine IL-6 and TNF-α cytokine concentrations using IL-6 and TNF-α ELISA kits according to the manufacturer’s instructions (Beyotime, Shanghai, China). The absorbance at 450 nm was detected with a microplate spectrophotometer.

### Western blot

RAW264.7 macrophages (6 × 10^5^ cells/well) were seeded into 6-well plates and incubated overnight (5% CO_2_, 37 °C). Cells were pretreated with with daidzin (50 µM), daidzein (50 µM) and dexamethasone (DXMS, 5 µM, positive control) for 4 h and then exposed to LPS (1 µg/mL) for 12 h to stimulated cells. Cells of different treatment groups were lysed with RIPA solution with 1% phenylmethanesulfonyl fluoride (PMSF) and 1% Protease Inhibitor Cocktail (Beyotime Biotechnology) on ice. The cell lysates were harvested and centrifuged at 15,000×*g* for 30 min at 4 °C to collect supernatants where the protein contents were measured by BCA Protein assay kit (Beyotime Biotechnology). Protein samples (15 µg) were separated by 8–10% SDS/PAGE gels and the resolved proteins were transferred to PVDF membranes (0.45 μm, Millipore, Billerica, MA, USA). The membranes were blocked with 5% non-fat milk in Tris-buffered saline Tween (TBST) for 2 h and incubated with appropriate primary antibodies overnight at 4 °C, where all of the primary antibody dilutions were 1:1000. The membranes were then incubated with the corresponding secondary antibodies diluted as 1:1000 for 2 h at room temperature. Finally, the proteins were detected with enhanced chemiluminescence (ECL) reagent (GE Healthcare, USA) and ChemiDoc MP Imaging System (Bio-Rad Laboratories, Hercules, CA, USA).

### Immunofluorescence assay

RAW264.7 macrophages (3 × 10^5^ cells/well) were seeded into confocal dish and incubated overnight (5% CO_2_, 37 °C). Cells were pretreated with daidzin (50 µM), daidzein (50 µM) and DXMS (5 µM, positive control) for 4 h and then exposed to LPS (1 µg/mL) for 4 h to stimulate cells. After washing 3 times with 1× phosphate-buffered saline (PBS), the cells were fixed with 4% paraformaldehyde (PFA) at room temperature for 15 min. The residual PFA was washed away with PBS. 0.1% Triton X-100 was used to permeabilize the fixed cells for 10 min. The cells were blocked with 3% BSA for 30 min at room temperature and then incubated with the primary antibody for p65 (1:200) overnight at 4 °C, followed by a 1-h incubation with Alexa Fluor 488-labeled secondary antibody (1:100, Beyotime Biotechnology) at 37 °C incubator. After washing with PBS, the cell nuclei were stained by DAPI (Beyotime Biotechnology). The fluorescence images were captured by Leica TCS SP8 confocal laser scanning microscope.

### Statistical analyses

In this study, GraphPad Prism (GraphPad Software Inc., La Jolla, CA, USA) was used to conduct statistical analysis. All the values were shown as mean ± S.E.M. All experiments mentioned above were repeated at least three times. The differences among different groups were analyzed by Kruskal–Wallis H test and Dunn’s post hoc test. A *p* < 0.05 was considered statistically significant.

## Results

### Daidzin and daidzein exert no cytotoxicity on RAW264.7 macrophages

**Fig. 2:**
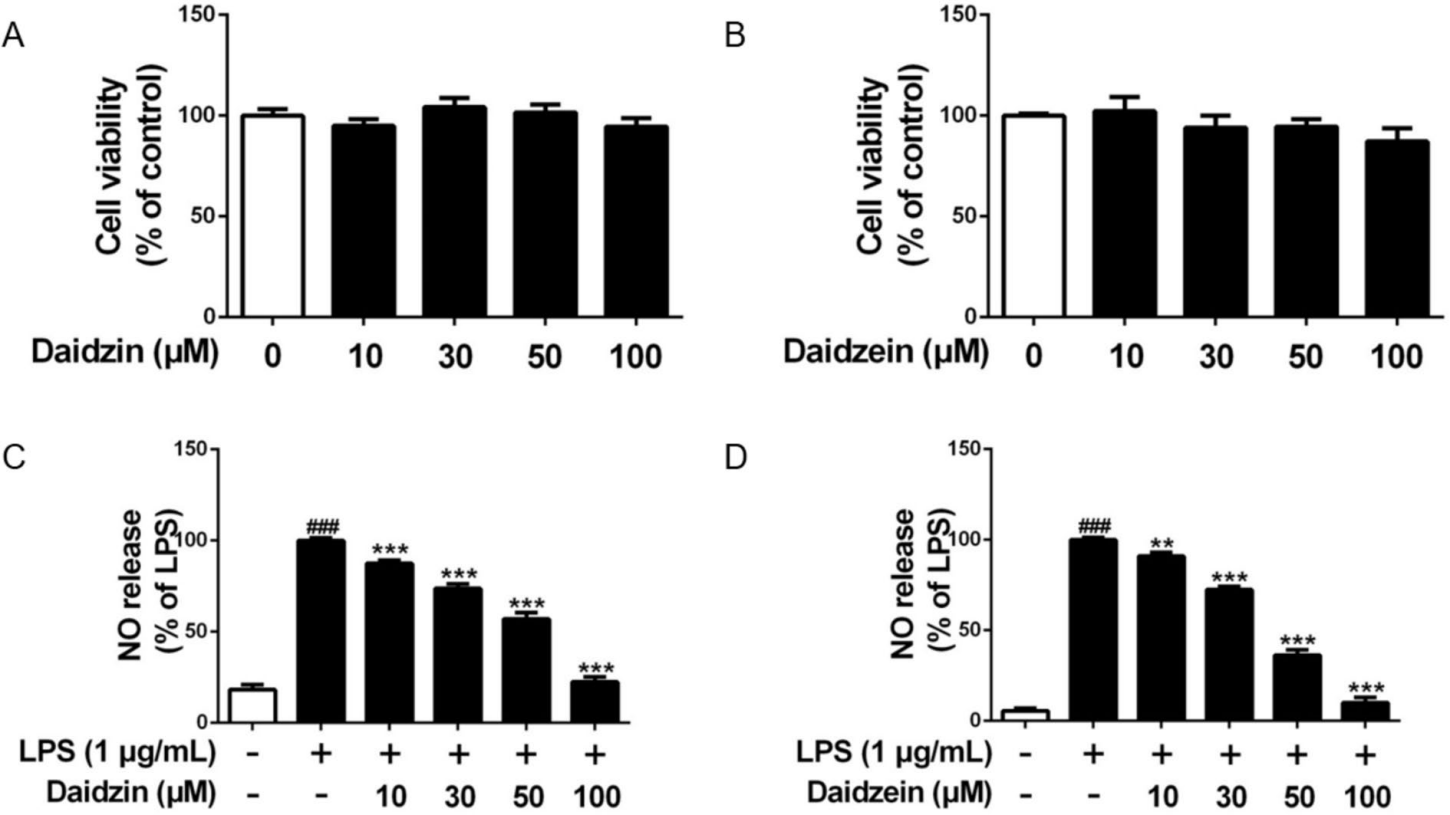
Effects of daidzin and daidzein on cell viability and NO release in the RAW264.7 macrophages. **A**, **B** The cell viability after treated with different concentrations of daidzin and daidzein for 24 h. **C**, **D** NO release in the RAW264.7 macrophages pretreated with daidzin and daidzein for 4 h and co-treated with LPS (1 µM) for another 12 h. Values are the means ± SEM (n = 3); **p < 0.01, ***p < 0.001, daidzin vs. LPS and daidzein vs. LPS; ^###^*p* < 0.001, LPS vs. Control

The cytotoxicity of daidzin and daidzein on RAW264.7 macrophages at different concentrations (10, 30, 50 and 100 µM for 24 h) were measured by MTT assay. Daidzin (Fig. 2A) and daidzein (Fig. 2B) did not have any observable effect on the cell viability of RAW264.7 macrophages within the contraction range being tested. Therefore, this series of non-toxic concentrations (10, 30, 50 and 100 µM) of daidzin and daidzein were selected for further studies on the anti-inflammatory effects and underlying mechanisms.

### Daidzin and daidzein inhibit NO production

As elucidated by Griess reagents kit, LPS (1 µg/mL, 12 h) markedly induced NO release in RAW264.7 cells; and the enhanced NO level was dose-dependently suppressed by daidzin (Fig. 2C) and daidzein (Fig. 2D). Daidzin and daidzein at high concentration (100 µM) reversed the LPS-triggered NO release to a level comparable to control whilst lower concentrations also showed inhibitory effects.

### Daidzin and daidzein reduce the secretions of inflammatory cytokines

**Fig. 3:**
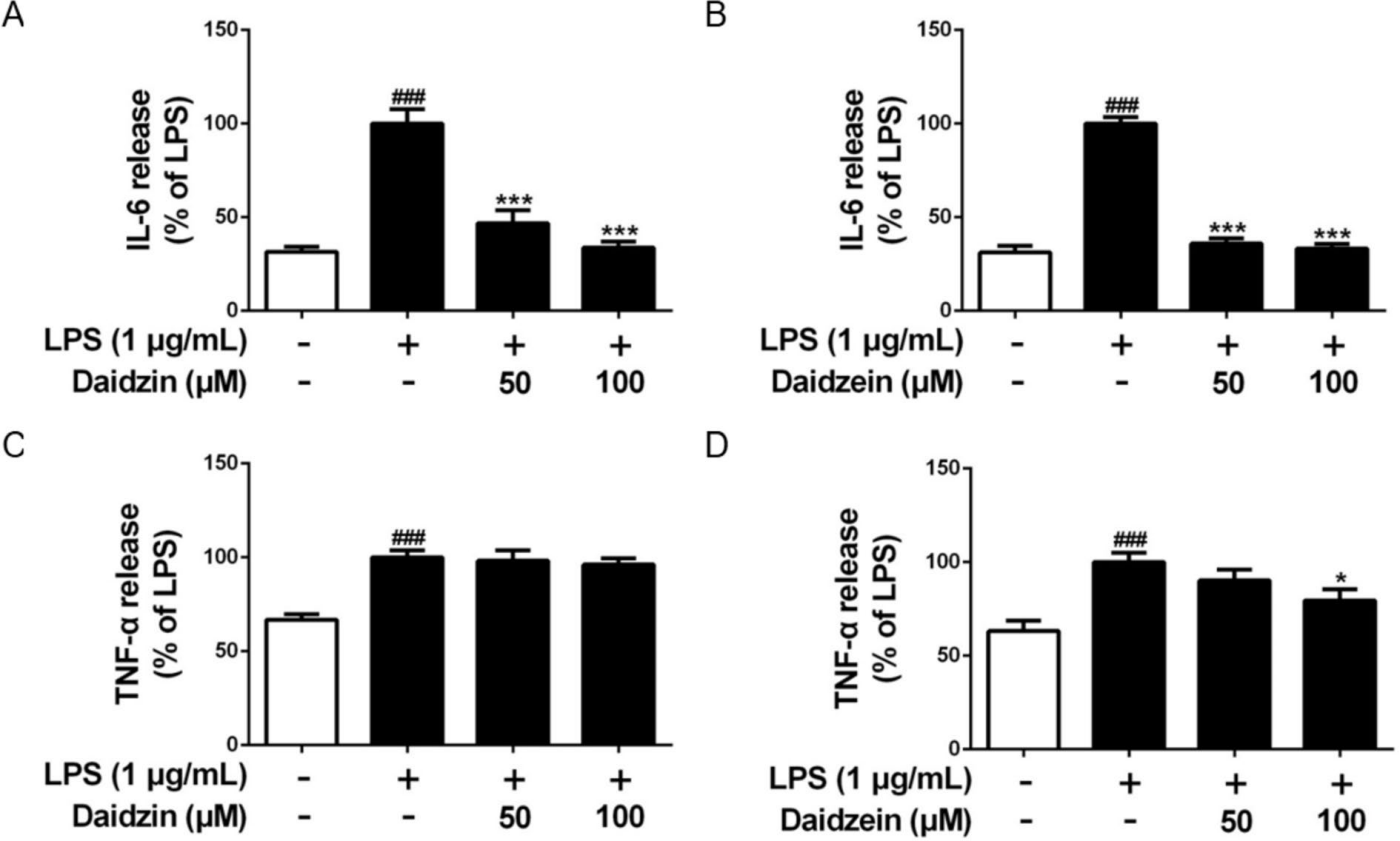
Effects of daidzin and daidzein on IL-6 and TNF-α in the RAW264.7 macrophages. **A**, **B** IL-6 and **C**, **D** TNF-α production in the RAW264.7 macrophages pretreated with daidzin and daidzein (50 and 100 µM) for 4 h and co-treated with LPS (1 µM) for another 12 h. Values are the means ± SEM (n = 3); *p < 0.05, ***p < 0.001, daidzin vs. LPS and daidzein vs. LPS; ^###^*p* < 0.001, LPS vs. control

The IL-6 and TNF-α cytokine levels were elucidated by corresponding ELISA kit to investigate the anti-inflammatory activity of daidzin and daidzein. The potent concentrations at 50 µM and 100 µM of these two compounds significantly reduced the release of IL-6 in the LPS-stimulated RAW264.7 macrophages (Fig. 3A, B). However, LPS-triggered TNF-α secretion was not reversed by daidzin under the same conditions but was moderately suppressed by daidzein at 100 µM (Fig. 3C, D). The results indicated that the inhibitory effect on IL-6 production is more effective and that TNF-α is likely a non-specific target of daidzin.

### Daidzin and daidzein suppress the expressions of inflammatory mediators in macrophages

**Fig. 4:**
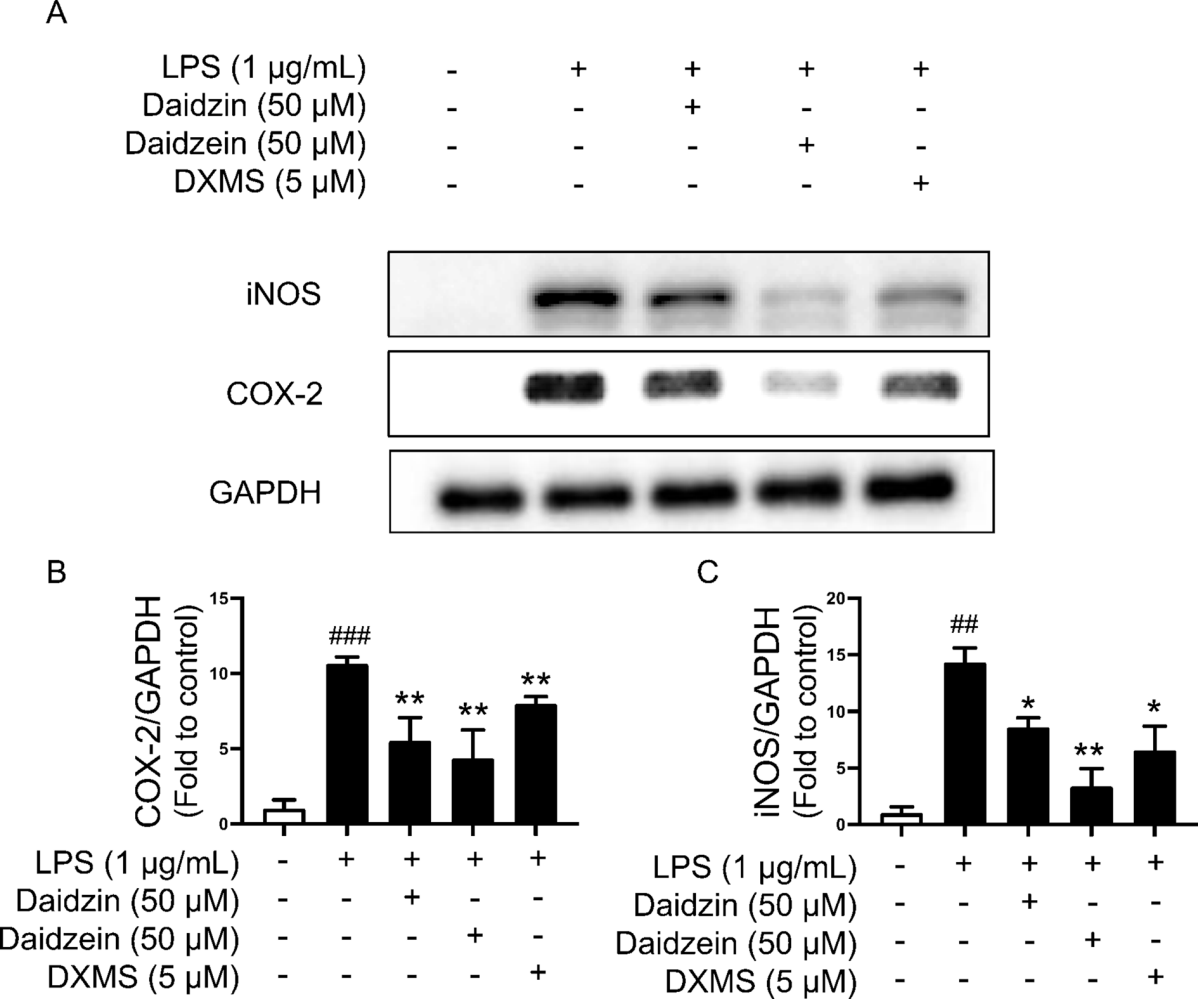
Effects of daidzin and daidzein on iNOS and COX-2. **A** Representative Western blots, and **B**, **C** summarized data for protein expressions of iNOS and COX-2 in RAW264.7 cells pretreated with daidzin (50 µM), daidzein (50 µM) and DXMS (5 µM, positive control) for 4 h and co-treated with LPS (1 µM) for another 12 h. Values are the means ± SEM (n = 4); *p < 0.05, **p < 0.01, daidzin vs. LPS and daidzein vs. LPS; ^##^*p* < 0.01, ^###^*p* < 0.001, LPS vs. control

Western blotting assay was performed to study the underlying mechanisms of the anti-inflammatory activities of daidzin and daidzein. Inducible nitric oxide synthase (iNOS) and cyclooxygenase-2 (COX-2) have been recognized as inflammatory mediators whilst MAPK and NF-κB signaling pathways play important roles in inflammation. Therefore, their protein expressions were determined. Stimulating RAW264.7 macrophages with LPS (1 µg/mL) for 12 h up-regulated the protein expressions of iNOS and COX-2 as compared with the control and such up-regulations were reversed by 4 h-pretreatment of daidzin (50 µM) and daidzein (50 µM) (Fig. 4A–C). The positive control DXMS also suppressed iNOS and COX-2 expressions.

### Daidzin and daidzein inhibit MAPK pathways

**Fig. 5:**
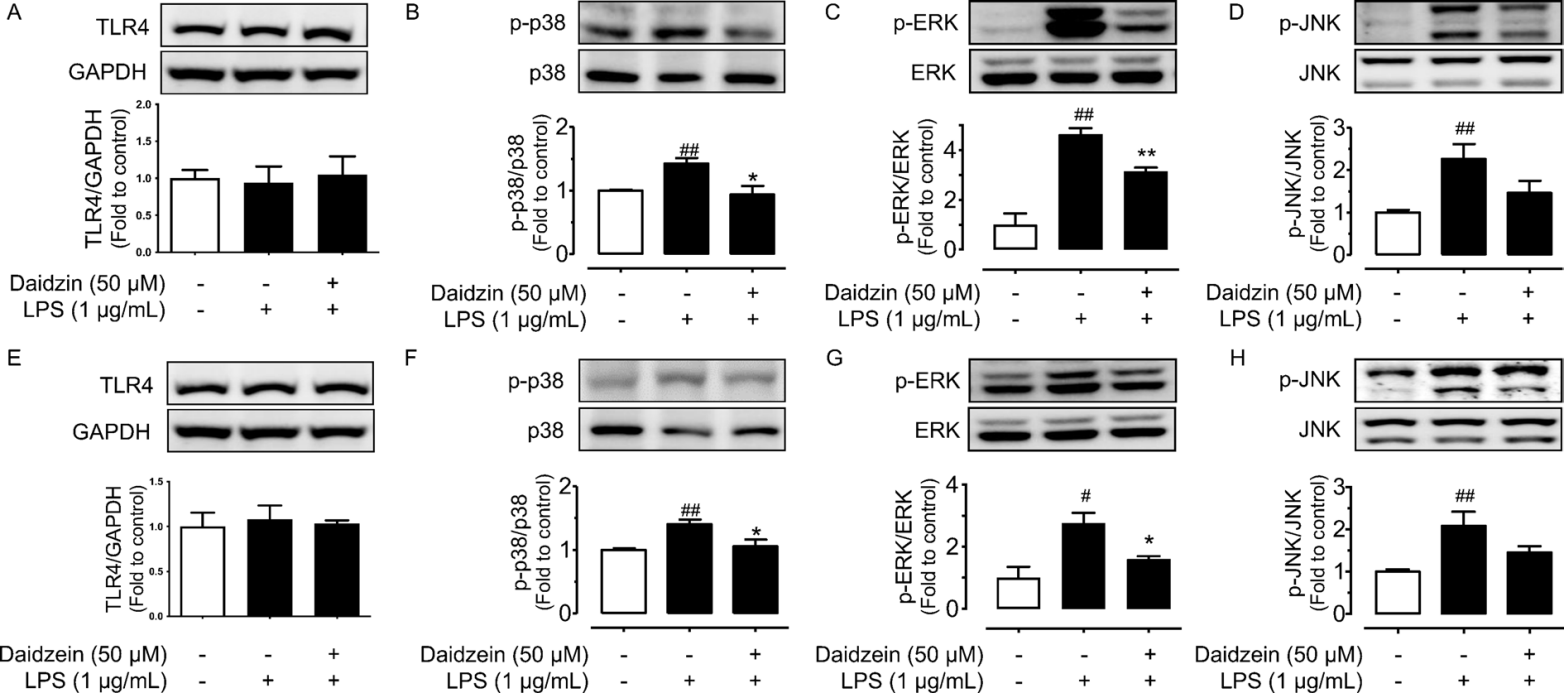
Effects of daidzin and daidzein on MAPK signaling pathways. **A** TLR4, **B** phosphorylation of p38 at Thr180/Tyr182, **C** ERK at Thr202/Tyr204, and **D** JNK at Thr183/Tyr185 normalized to its corresponding total protein in RAW264.7 cells pretreated with daidzin (50 µM) for 4 h and co-treated with LPS (1 µM) for another 12 h. **E**–**H** Protein expression of TLR4, phosphor-p38, phosphor-ERK and phosphor-JNK for daidzein treatment (50 µM) with LPS. Values are the means ± SEM (n = 4); *p < 0.05, **p < 0.001, daidzin vs. LPS and daidzein vs. LPS; ^#^*p* < 0.05, ^##^*p* < 0.01, LPS vs. control

The expressions of toll-like receptor 4 (TLR4) which specifically recognizes LPS were not altered in different treatment groups (Fig. 5A, E). MAPK pathways were activated when macrophages were stimulated with LPS (1 µg/mL, 12 h): phosphorylation levels of p38 at Thr180/Tyr182, ERK at Thr202/Tyr204 and JNK at Thr183/Tyr185 were upregulated without altering the total protein expressions. Pre-incubation with daidzin (Fig. 5B–D) or daidzein (Fig. 5F–H) at 50 µM was effective to suppress the phosphorylation of p38 and ERK. However, both compounds showed minor but not significant inhibition of JNK phosphorylation.

### Daidzin and daidzein inhibit NF-κB signaling pathways

**Fig. 6:**
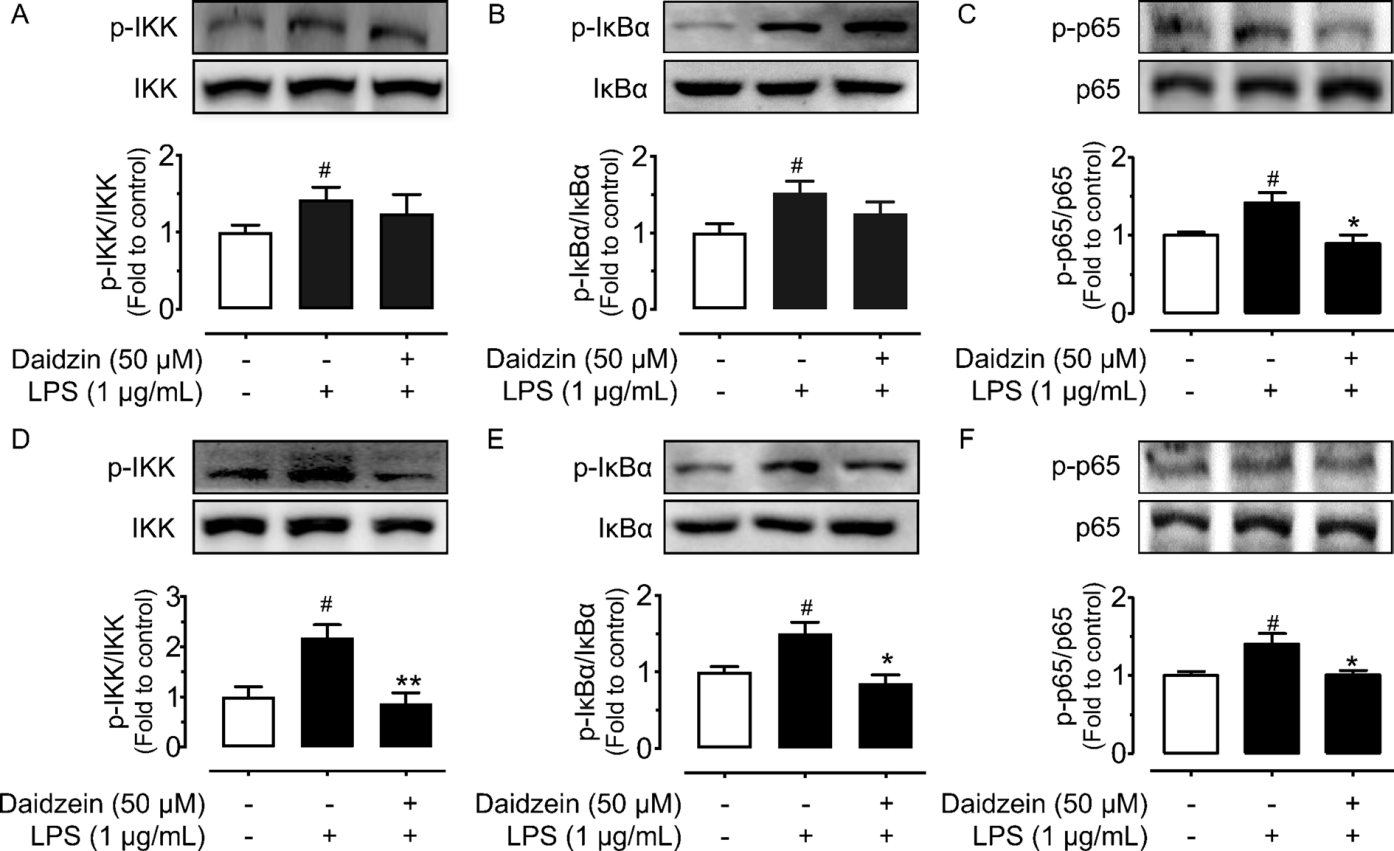
Effects of daidzin and daidzein on NF-κB signaling pathway. **A** Phosphorylation of IKKα/β at Ser176/180, **B** IκBα at Ser32, and **C** p65 at Ser536 normalized to its corresponding total protein in RAW264.7 cells pretreated with daidzin (50 µM) for 4 h and co-treated with LPS (1 µM, 12 h). **D**–**F** Protein phosphorylation of IKK, IκBα and p65 for daidzein treatment (50 µM) with LPS. Values are the means ± SEM (n = 4); *p < 0.05, **p < 0.01, daidzin vs. LPS and daidzein vs. LPS; ^#^*p* < 0.05, LPS vs. control

**Fig. 7:**
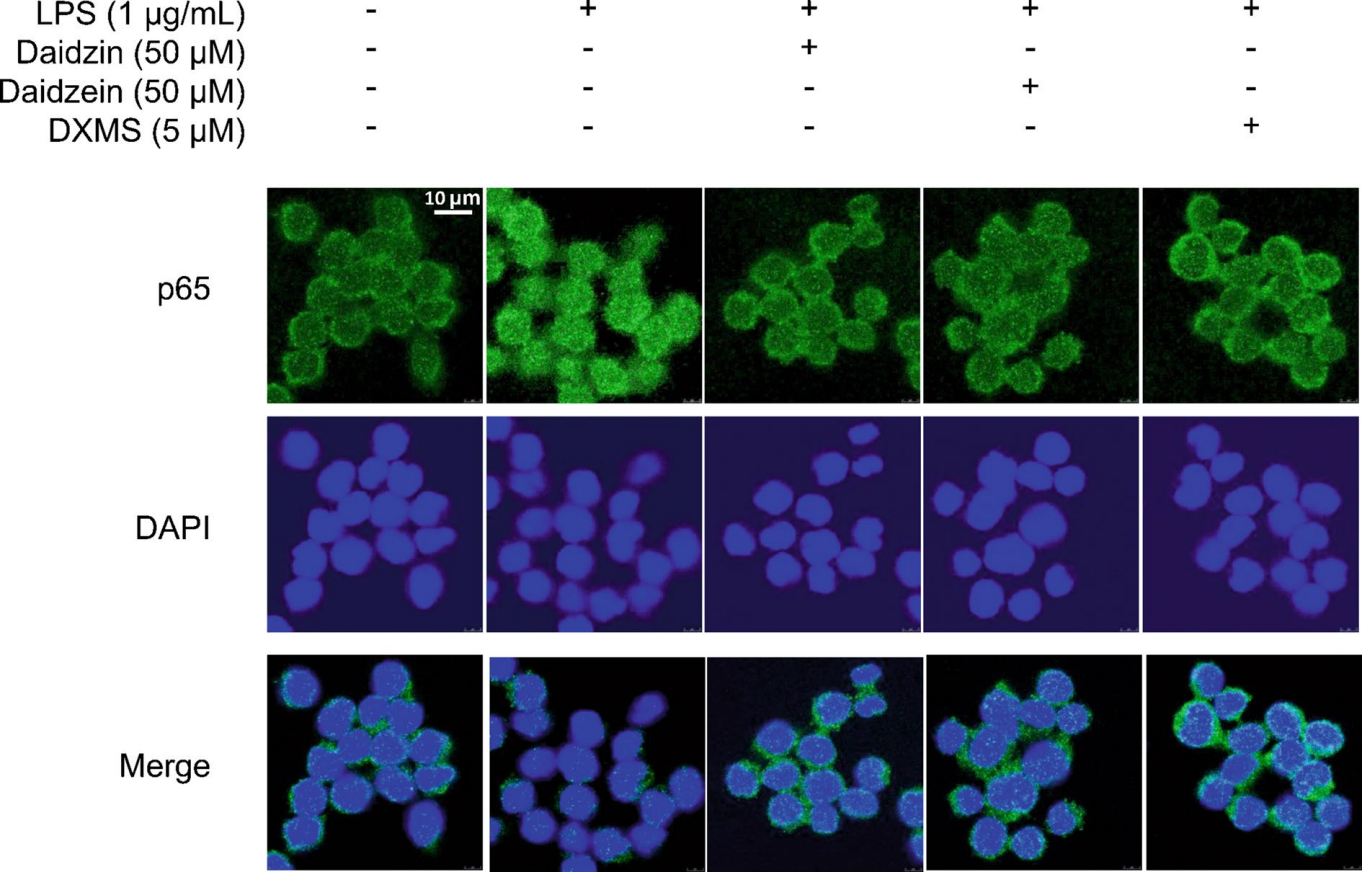
Effects of daidzin and daidzein on nuclear translocation of p65 subunit of NF-κB. Representative images of p65 nuclear translocation in RAW264.7 cells pretreated with daidzin, daidzein and DXMS (positive control) for 4 h and co-treated with LPS for another 4 h. Four independent experiments were performed

Likewise, exposure of macrophages to LPS (1 µg/mL, 12 h) activated NF-κB signaling pathways, increasing phosphorylation of IKKα/β at Ser176/180, IκBα at Ser32, and p65 at Ser536 as normalized to its respective total protein. Daidzein pretreated at 50 µM only reduced phosphorylation of p65 at Ser536 but not IKKα/β or IκBα in LPS-triggered cells (Fig. 6A–C); whereas daidzein reversed the increase of phosphorylation for IKKα/β, IκBα and p65 (Fig. 6D, E). In addition, the nuclear translocation of p65 subunit of NF-κB was increased by LPS treatment in RAW264.7 cells. These translocations were markedly inhibited by pretreatment of daidzin (50 µM), daidzein (50 µM) and DXMS (5 µM), as detected by immunofluorescence assay (Fig. 7). The results supported that daidzin and daidzein ameliorate LPS-induced inflammation potentially via the inactivation of MAPK and NF-κB signaling pathways.

## Discussion

Our hypothesis was supported by the results. The present work showed that two soy isoflavones, daidzin and daidzein, exhibited dose-dependent manner of anti-inflammatory activities in LPS-stimulated RAW264.7 macrophages. The anti-inflammatory activities of daidzin and daidzein were supported by the findings: (1) reducing NO release; (2) inhibiting secretions of inflammatory cytokines (significantly for IL-6 but mildly for TNF-α); and (3) down-regulating the expression of inflammatory indicators iNOS and COX-2. Additionally, the effect was mediated by suppressing ERK/p38 MAPK and NF-κB p65 pathways.

According to the earlier studies, LPS-induced RAW264.7 macrophages are commonly used as models of inflammation [25, 26]. In inflammatory response, vast quantities of NO are synthesized by iNOS that is induced by activated immune cells and LPS [27, 28]. Therefore, NO and iNOS are generally recognized as inflammatory indicators. COX-2 is an inflammatory modulator and an enormous amount of COX-2 can be found in the inflammatory sites [29]. Dexamethasone, a corticosteroid, is similar to a natural hormone produced by adrenal glands. DXMS relieves inflammation in various parts of the body as an anti-inflammatory medication and thus was used as positive control in present study [30–33]. In this study, daidzin and daidzein can effectively suppress these inflammatory indicators including NO production and the expressions of iNOS and COX-2 to levels comparable with the positive control, supporting both compounds can ameliorate the LPS-induced inflammation in the RAW264.7 macrophages. These results are consistent with the preceding studies [22, 23]. However, the underlying mechanism, particularly for daidzin, remains largely unknown and needs further investigation.

TLR4 is known to mediate signal transduction of LPS and activate inflammation pathways such as MAPK and NF-κB signaling pathways [34, 35]; nevertheless, protein expression of TLR4 had no significant difference among different groups in present study. Both MAPK and NF-κB signaling pathways play the important roles in the inflammation. MAPKs involve three serine-threonine kinases: extracellular signal-regulated kinase (ERK), c-Jun NH(2)-terminal kinase/stress-activated protein kinase (JNK/SAPK) and p38 MAPK. NF-κB is a family of inducible transcription factors, modulating the gene transcriptions responsible for inflammation [36]. Of note, there is a cross-talk of MAPK and NF-κB signaling pathways: transcriptional activity of NF-κB can be regulated by MAPK signaling cascades [37]. Activation of MAPK and NF-κB signaling pathways promotes protein expression of iNOS and COX-2 and the release of pro-inflammatory cytokines, such as TNF-α, IL-1 and IL-6; whereas inhibition of these pathways can suppress inflammatory responses [38–42].

TNF-α and IL-6 both can modulate immune responses and are involved in numerous pathologic and physiologic processes. They are linked to several diseases such as cancers and diabetes, and worsen inflammation [43–45]. Here we found that daidzin and daidzein remarkably reduced the IL-6 level stimulated by LPS but only exerted minor effect on TNF-α production. Daidzin did not affect TNF-α release while daidzein at high concentration (100 µM) moderately diminished TNF-α level. Previous studies have demonstrated that significantly daidzein decreased the IL-1β level and increased IL-10 in the serum and kidney tissue to improve inflammation in ovariectomized rats, and also up-regulated transforming growth factor (TGF)-β to stimulate collagen synthesis [46, 47]. However, the current study cannot rule out the contribution of other pro-inflammatory cytokines like IL-1β and/or anti-inflammatory cytokines such as IL-10 and TGF-β. Future extensive efforts are still needed to explore the effects of daidzin and daidzein on the generation of various cytokines.

Previous studies have demonstrated that daidzein reduces inflammation in LPS-stimulated macrophages via inhibiting STAT-1 and NF-κB activations [22, 23, 48]; and a recent study with another model using LPS-induced hepatocytes has also shown that daidzein inhibits NF-κB signaling cascade [19]. In line with previous results, we found that daidzein suppressed the nuclear translocation and NF-κB p65 activation to decrease the gene transcriptions responsible for inflammation; and our novel findings indicated that daidzein inhibited ERK/p38 MAPK as well. On the other hand, the mechanism for the anti-inflammatory effect of daidzin was unexplored [24]. The current study supported that daidzin exhibited similar potency to suppress inflammation in RAW264.7 cells as its aglycone daidzein, inhibiting both p38/ERK MAPK and NF-κB p65. The effective concentrations were similar for both isoflavones. Daidzein showed some additional effects to reduce the phosphorylation of IKKα/β and IκBα to moderately inhibit TNF-α production. However, these were unaffected by daidzin at the tested concentrations.

In China, *Radix Puerariae* is widely used as traditional Chinese medicine [49, 50] and consumed as food in daily life. Anti-inflammation is one of the beneficial effects of *Radix Puerariae*, which may be attributed to its active ingredients daidzin and daidzein as revealed by the current findings. This study provided evidence to support the anti-inflammatory effect of daidzin and daidzein in the LPS-induced RAW264.7 macrophages, which was mediated through inhibition of the MAPK and NF-κB signaling pathways. The anti-inflammation effects of daidzin and daidzein should be further investigated in the future *in vivo.* Furthermore, the potential targets of daidzin and daidzein, like TLR4, p65 and so on, should be explored in the future by performing molecular docking assay or inhibition assays to predict the compound-targeting interaction.

## Conclusion

In conclusion, our data from the in vitro investigations illustrate that daidzin and daidzein can inhibit LPS-induced inflammation to significantly suppress NO and IL-6 level by regulating MAPK and NF-κB signaling pathways in RAW264.7 cells. Daidzin and daidzein as well as the food and herbs containing them like soy products and *Radix Puerariae* may have therapeutic potential for inflammatory diseases.

## Data Availability

Not applicable.

## Abbreviations

COX-2: Cyclooxygenase-2
IL: Interleukin
iNOS: Inducible nitric oxide synthase
LPS: Lipopolysaccharides
MAPK: Mitogen-activated protein kinase
MTT: 3-(4,5-Dimethylthiazol-2-yl)-2,5-diphenyltetrazolium bromide
NF-κB: Nuclear factor kappa B
NO: Nitric oxide
TLR4: Toll-like receptor 4
TNF-α: Tumour necrosis factor-α
